# Diagnostic Accuracy of Magnetic Resonance Cholangiopancreatography in Comparison With Endoscopic Retrograde Cholangiopancreatography for Detection of the Etiology of Obstructive Jaundice

**DOI:** 10.7759/cureus.34484

**Published:** 2023-02-01

**Authors:** Javaria Isram, Ehtesham Haider, Rao Saad Ali Khan, Muhammad Hafeez, Rashk e Hinna, Isfandyar Baig, Aqsa Shahid, Manahil Chaudhry

**Affiliations:** 1 Gastroenterology, Pak Emirates Military Hospital (PEMH), Rawalpindi, PAK; 2 General Medicine, Royal Bournemouth Hospital, Bournemouth, GBR; 3 General Medicine, The Mid Yorkshire Hospital NHS Trust, Wakefield, GBR

**Keywords:** choledocholithiasis, common bile duct (cbd), endoscopic retrograde cholangiopancreatography (ercp), obstructive jaundice, magnetic resonance cholangiopancreatography (mrcp)

## Abstract

Background: Despite technological advances, obstructive jaundice has significant morbidity and mortality rates. When studying obstructive jaundice, endoscopic retrograde cholangiopancreatography (ERCP), the "gold standard" for biliary obstruction identification, might be replaced with magnetic resonance cholangiopancreatography (MRCP), which is a non-invasive procedure.

Objective: Diagnostic accuracy of MRCP in comparison with ERCP for the detection of the etiology of obstructive jaundice.

Methodology: This prospective, observational study included 102 patients who presented with obstructive jaundice as proven by liver function tests. The MRCP was conducted within 24 to 72 hours before the ERCP. A torso phased-array coil (Siemens, Germany) was used for the MRCP. The duodeno-videoscope and general electric fluoroscopy were used to perform the ERCP. The MRCP was evaluated by a classified radiologist who was blinded to the clinical details. An experienced consultant gastroenterologist who was blinded to the results of the MRCP assessed the cholangiogram of each patient. The hepato-pancreaticobiliary system results from both procedures were compared based on the pathology observed, such as choledocholithiasis, pancreaticobiliary strictures, and dilatation of biliary strictures. We determined the sensitivity, specificity, and negative and positive predictive values with 95% confidence intervals. The statistical significance was set at p<0.05.

Results: The most commonly reported pathology was choledocholithiasis, and MRCP diagnosed 55 patients, of which 53 were true positive cases when compared with the ERCP results of the same patients. MRCP demonstrated greater sensitivity and specificity (respectively) for screening choledocholithiasis (96.2, 91.8), cholelithiasis (100, 75.8), pancreatic duct stricture (100, 100), and hepatic duct mass (100, 100) and showed statistically significant values. The sensitivity of MRCP is lower for identifying benign and malignant strictures, but its specificity was observed to be reliable.

Conclusion: When it comes to determining the severity of obstructive jaundice, both in its early and later stages, the MRCP technique is widely regarded as a reliable means of diagnostic imaging. The diagnostic function of ERCP has been significantly reduced as a result of the precision of MRCP as well as its non-invasive nature. In addition to being a helpful non-invasive method to identify biliary diseases and avoid unnecessary ERCPs and their risks, MRCP offers good diagnostic accuracy for obstructive jaundice.

## Introduction

Obstructive jaundice is characterized by an increase in serum bilirubin levels, and this might be the result of a partial or complete blockage of the drainage of bile into the second part of the duodenum from the liver, which eventually leads to cholestasis [1]. Obstructive jaundice is the clinical manifestation of cholestasis, which may develop in the ductule system of the liver (hepatic cholestasis) or in the extrahepatic bile duct system owing to mechanical blockage (obstructive jaundice or extrahepatic cholestasis). Obstructive jaundice is not a diagnostic confirmation; a timely examination to determine the origin of cholestasis is crucial since symptoms may develop if the blockage is not eased [2]. These include yellowing of the skin and sclera, itching, dark-coloured urine, and fatigue.

Serum biochemical parameters validate the confirmation of jaundice with a high serum total bilirubin concentration, typically >40 µmol/l when clinically noticeable. The other liver function tests reveal an obstructive pattern, including a more than five times elevation in alkaline phosphatase with a moderate rise in the levels of transaminases [3]. The bilirubin level may also be raised. Cholestasis may be extrahepatic or intrahepatic and is usually accompanied by an altered level of prothrombin time along with biochemical abnormalities in liver function tests [4]. Information regarding treatment options is crucial for physicians to make management and therapy decisions. Yet, in the span of the last few years, there have been considerable breakthroughs in our knowledge of the pathophysiology, diagnosis, staging, and effectiveness of treatment for obstructive jaundice [5]. However, due to the widening range of treatment options for jaundice, radiologists are now expected to use more than one screening tool to rule out obstructive jaundice. However, an accurate diagnosis of the disease's cause, location, severity, and progression is crucial for selecting the best course of treatment.

Endoscopic retrograde cholangiopancreatography (ERCP) is considered the "gold standard" diagnostic tool for biliary blockage detection and is also known as one of the few methods of direct invasive cholangiography. However, it is an imprecise diagnostic tool, and in the future, alternative methods may be better-suitable gold standards for diagnosis [6,7]. Magnetic resonance imaging, or MRI, has been called the most defining step forward in medical diagnosis and has become a possible new imaging method as quick imaging sequences, specific surface coils, and better image quality have been developed [8]. Magnetic resonance cholangiopancreatography (MRCP) is a relatively advanced emerging MRI method that allows for a noninvasive assessment of the biliary system in individuals suspected of having obstructive jaundice [9]. For situations where an ERCP cannot be performed due to anatomical or technical constraints, MRCP should be used instead. Moreover, MRCP is capable of producing projectional pictures of the pancreatic duct and the biliary tree in addition to providing excellent cross-sectional views of ductal characteristics [10]. Invasive ERCP surgery has a number of side effects, including the risk of developing an infection, pancreatitis, haemorrhage, etc. [11]. In contrast to an ERCP, an MRCP is non-invasive and does not require any oral or intravenous contrast [12].

The advantages of ERCP include the ability to treat the cause of jaundice and take a biopsy of the lesion, but the benefits of MRCP cannot be overlooked as it offers great patient acceptability along with decreased levels of discomfort and increased levels of safety. Presently, it is thought that MRCP and ERCP are equally accurate in diagnosing a range of biliary duct and pancreatic diseases, whether they are malignant or benign in nature. MRCP is comparable to diagnostic ERCP for visualizing the biliary tree and determining biliary blockage. In contrast to ERCP, which is used for both therapeutic and diagnostic purposes, MRCP does not provide therapeutic options. When MRCP is used to diagnose a biliary blockage, invasive procedures such as diagnostic ERCP may be avoided [13].

## Materials and methods

Patient recruitment

Patients with obstructive jaundice who underwent MRCP at the gastroenterology department of the Pak Emirates Military Hospital (PEMH), Rawalpindi (Pakistan) from December 1, 2021 until September 1, 2022, were included in this study after informed written consent. This prospective trial included 102 individuals with probable pancreatobiliary illness (manifested as obstructive jaundice) who underwent an MRCP followed by an ERCP. When assessing the MRCP, the radiologist did not have access to the outcomes of the other imaging modalities. Patients with serious health problems that would prevent them from undergoing the MRCP procedure were not included in the study (e.g., claustrophobia, ankylotic or degenerative spine disorders, cardiac pacemakers, etc.). In addition to that, patients who had a deranged coagulation profile (international normalized ratio [INR] >1.5), were haemodynamically unstable, had a low platelet count (<50,000/mm3), low haemoglobin (Hb <7) or were unfit for anaesthesia were excluded from the study.

MRCP technique

For MRCP, a torso-phased array coil (Siemens, Germany) was utilized. MRCP sequence planning uses three-plane gradient-echo localizing images. Single-shot fast spin-echo (SSFSE) sequences were used to take axial slices with the following specifications: 2.1 TE, 28-38 cm field of view, 7 mm slice thickness, 1-2 mm spacing, and 256 kHz frequency. Radial slice acquisitions with high resolution, thick slabs, and long TE were made in the area of the biliary and pancreatic conduits. We made use of 12 reconstructed slices, spaced 10 degrees apart. After a 12-hour fast, all of the sequences were recorded during a single breath hold to promote gallbladder filling. A consultant radiologist and his team, who only had clinical knowledge of the patient's symptoms, assessed the MRCP data. The contrast was used where the suspicion of malignancy was high or was clinically indicated.

ERCP technique

**Figure 1:**
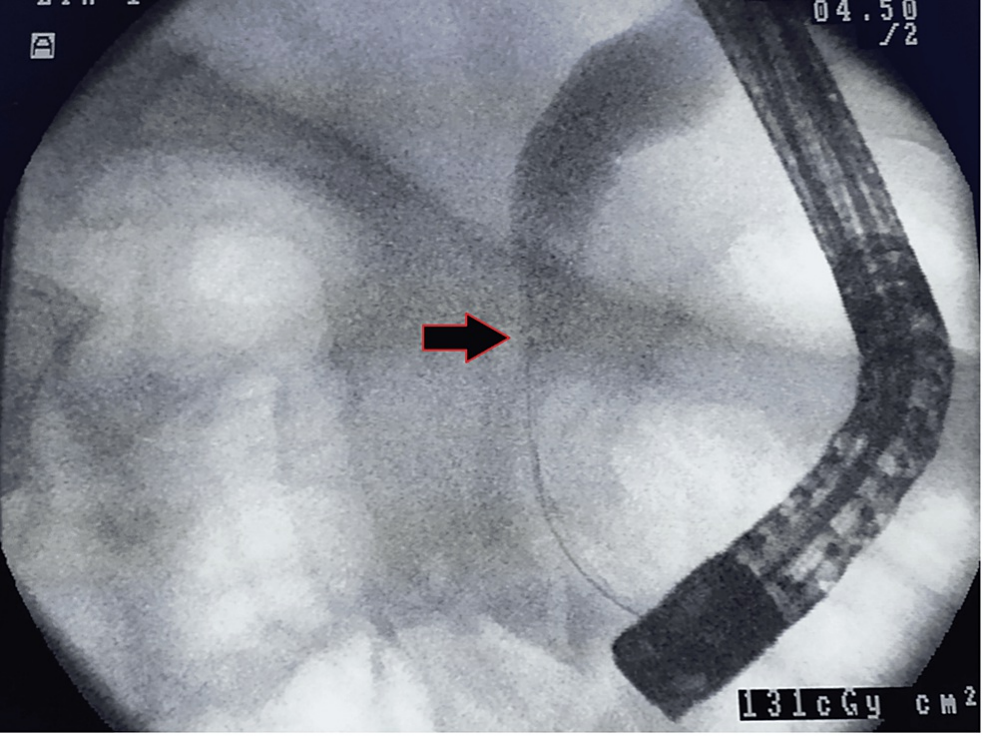
Endoscopic retrograde cholangiopancreatography image demonstrating a stricture (arrow) with upstream dilatation of the common bile duct.

ERCP was performed utilizing a duodeno-videoscope and general electric fluoroscopy from an Olympus JF type 230 (Olympus, Japan) while patients were under conscious sedation or general anaesthesia as suggested by anaesthesia team. The ERCP was performed on prone patients by a qualified gastroenterologist who had no access to the results of the prior MRCP. ERCP allowed for the collection of tissue biopsies and bile cytology for further diagnosis of benign versus malignant findings. On the basis of the pathophysiology of the biliary system, the outcomes of both procedures were compared and assessed (Figure 1).

Analytical statistics

The two imaging approaches were compared using their specificity, sensitivity, and negative and positive predictive values. Fisher's exact 2 × 2 tests with 95% confidence intervals were used to perform group comparisons, with a p-value < 0.05 considered statistically significant.

## Results

A total of 102 participants were enrolled in this study with an age range of 7 years to 93 years, and the reported mean age was 53.48 + 16.90. The mean values of total bilirubin, alanine transaminase, and alkaline phosphatase were 63.7 + 40.61 µmol/L, 111.96 + 82.90 U/L, and 310.82 + 142.40 U/L, respectively. The majority of them were male 55.9% (57) and 44.1% (45) of them were female (Table 1).

**Table 1:** Descriptive statistics All descriptive data were presented in mean + SD. Gender information was calculated in frequency and percentage. SD: standard deviation.

	Minimum	Maximum	Mean + SD
Age (years)	7	93	53.4 + 16.901
Alanine transaminase (U/L)	20	456	111.96 + 82.901
Alkaline phosphatase (U/L)	114	906	310.82 + 142.40
Total bilirubin level (µmol/L)	13	233	63.70 + 40.614
Gender	Frequency	Percentage (%)	
Female	45	44.1	
Male	57	55.9	

As per our study findings, MRCP showed a higher sensitivity for the screening of masses at the pancreatic head causing common bile duct (CBD) stricture, cholelithiasis, and a mass at the confluence of the hepatic duct and main pancreatic duct stricture, whereas higher specificity was reported for modalities like malignant distal CBD stricture, main pancreatic duct stricture, and mass at the confluence of the hepatic duct (Table 2).

**Table 2:** Magnetic resonance cholangiopancreatography: sensitivity, specificity and predictive values (n=102) The sensitivity, specificity, positive predictive values and negative predictive values using magnetic resonance cholangiopancreatography were calculated for diagnostic modalities of obstructive jaundice. Data were presented as percentages. CBD: common bile duct.

Clinical characteristics	Sensitivity (%)	Specificity (%)	PPV (%)	NPV (%)
Choledocholithiasis	51 (96.2)	45 (91.8)	92.7	95.7
Cholelithiasis	11 (100)	69 (75.8)	33.3	100
Malignant distal CBD stricture	2 (15.4)	89 (100)	2100	89
Benign distal CBD stricture	1 (4.3)	77 (97.5)	33.3	77.8
Mass pancreatic head causing CBD stricture	8 (100)	92 (97.9)	80	100
Main pancreatic duct stricture	3 (100)	99 (100)	100	100
Mass at confluence of hepatic duct	11 (100)	91 (100)	100	100

Choledocholithiasis was the most common ERCP diagnosis (53 patients), and MRCP diagnosed it in 55 cases. There were a total of eight cases of distal CBD stricture, out of which MRCP diagnosed six false positive results. In our study group, we had 23 benign and 13 malignant cases of distal CBD strictures. MRCP correctly diagnosed only three benign and two malignant CBD stricture cases. The MRCP successfully screened all cases of pancreatic duct stricture and mass of the hepatic duct. MRCP demonstrated 10, of which eight were actually pancreatic head masses, i.e., two cases were false positives (Table 3).

**Table 3:** Comparison of ERCP Vs MRCP diagnostic outcomes in obstructive jaundice (n=102) The diagnostic modalities of obstructive jaundice were compared between ERCP and MRCP. Fisher’s 2 × 2 exact test was performed to compare their outcomes. P-value <0.05 was considered significant with the confidence intervals of 95%. ERCP: endoscopic retrograde cholangiopancreatography, MRCP: magnetic resonance cholangiopancreatography, CBD: common bile duct.

Clinical characteristics	MRCP	ERCP	p-value
Choledocholithiasis	55	53	<0.05
Cholelithiasis	33	11	<0.05
Distal CBD stricture	14	8	0.595
Mass pancreatic head causing CBD stricture	10	8	<0.05
Main pancreatic duct stricture	3	3	<0.05
Mass at the confluence of hepatic duct	11	11	<0.05

## Discussion

The rise in popularity of MRCP may be attributed to the limitations of other imaging modalities as well as the benefits that it provides. Despite the fact that MRCP scans have certain benefits over ERCP, they are restricted in their capacity to appropriately determine the level and aetiology of blockage. As a result of this, invasive procedures like the ERCP are often necessary. Morbidity rates ranging from 1% to 7% have been linked to ERCP [14,15]. In addition, the rates of failed duct cannulation that have been observed range from 3% to 10% [14,16]. In recent years, MRCP has emerged as a fast-emerging method for the examination of biliary blockage. Through MRCP, it is possible to provide detailed projectional-type pictures that are comparable to direct cholangiography. It is generally not operator-dependent compared to ERCP, which is highly operator-dependent and does not need the administration of any intravenous contrast.

The primary benefit of MRCP in the diagnosis of biliary obstruction is that it allows for the routine viewing of ducts both above and below the blockage, as well as the reliable demonstration of intrahepatic biliary branches. MRCP is most effective for treating patients who have high strictures in their bile ducts as well as those who have a biliary blockage at numerous levels [16]. It is possible to visualize biliary ducts that have been excluded from the main duct system due to damage, and these ducts may be identified without difficulty during MRCP. Additionally, it is helpful in situations in which ERCP is not completely successful or when cannulation of the bile duct cannot be performed due to anatomical constraints or technological challenges [17]. In addition, MRCP is capable of providing a physiological assessment of the bile ducts, in contrast to ERCP, which has the potential to alter the real anatomic situation if either too much or too little contrast is used.

Recent research has shown that MRCP has a high degree of accuracy when it comes to determining both the extent and the aetiology of blockage. In a research study, blockage level was determined and biliary dilatation was detected in 87% of the patients [18]. Magnuson et al. found the level of obstruction in 24 of 25 patients with a malignant cause of the blockage, and they found the cause in 21 of those individuals. They were able to discover 45 individuals out of 48 who had causes that were treatable [19]. The obstruction level was also detected in some studies in 13 (87%) of 15 patients suffering from malignant blockage [20]. According to one research study finding, MRCP had a sensitivity of nearly 90% and a specificity that reached almost 100% when it came to identifying the existence of biliary obstruction as well as the severity of the obstruction.

In our study, malignant distal CBD stricture was correctly identified in only 2 cases out of 13 cases, and therefore it had 15.4% sensitivity and 100% specificity. MRCP was successful in identifying all the cases of pancreatic duct stricture (i.e., 3 cases) and hepatic duct mass (i.e., 11 cases) as per current findings. In a recent study, it was found that MRCP had a sensitivity of 94.44%, a specificity of 81.81%, a positive predictive value of 89.47%, and a negative predictive value of 90% for the detection of malignant obstruction [21].

As per our study findings, all instances of choledocholithiasis were detected by MRCP; however, there were two false positives, which was consistent with results from the 2001 and 1998 investigations [22,23]. While choledocholithiasis may be detected with 91% sensitivity and 98% specificity by MRCP, as a study reported in 1999 [24], cholelithiasis was also reported with higher sensitivity (100%) and specificity (75.8%) in our study findings (Table 2). Furthermore, gallstone diagnosis was more accurate with MRCP, as reported in one study [25].

It was found that the detection of bile duct strictures caused by chronic pancreatitis had a sensitivity and specificity of 75% and 69%, respectively [26]. Research that was carried out by Al-Obaidi et al. demonstrated that MRCP had greater sensitivity (100%), specificity (98.5%), and accuracy (98.7%) for cases with benign strictures, but in our case, sensitivity and specificity were 4.3% and 93.5% for benign CBD strictures [22]. A tiny pancreatic head mass was misinterpreted as cholangiocarcinoma in a study done by Vaishali and colleagues [21]. In the current study, MRCP correctly identified eight cases of pancreatic head mass, whereas two cases were misdiagnosed as false-positive results with high sensitivity (100%) and specificity (97.9%).

Indications for MRCP include contraindicated or unsuccessful ERCP; patient preference for non-invasive imaging; patients at low risk for biliary or pancreatic complications; patients for whom the therapeutic ERCP is almost impossible; and patients with suspected neoplastic etiology for biliary or pancreatic obstruction. Patient preparation needed for the MRCP is keeping the patient nil by mouth a few hours before the exam and breath holding during the exam. Sedation is not required in most cases. MRCP is especially effective in situations when ERCP is impossible, dangerous, or difficult. It is also a potential alternative for ERCP failure patients. The contraindications for MRCP and ERCP are distinct, enabling them to be used as complementary procedures [25].

In a variety of bile duct disorders (tumour, stricture, and blockage), MRCP has the ability to take on the role of diagnostic ERCP, preventing any potential need for surgery (unless required therapeutically) and associated risks. MRCP is a more accurate examination than diagnostic ERCP, according to current data; however, ERCP is still required for therapeutic purposes, even though it is still used to diagnose biliary disorders.

Study limitations

Because of the limited size of the research population, the results of the other biliary pathology tests were inconsistent. In the future, an additional study must be conducted with a greater number of patients in a different hospital setting in order to get the same conclusion. In some cases, patients were unable to hold their breath long enough, affecting the quality of the 3D MRCP sequence.

## Conclusions

MRCP exhibited a high level of diagnostic precision for obstructive jaundice. According to the most recent studies, MRCP has the potential to replace diagnostic ERCP in a wide variety of bile duct anomalies (bile stones, benign and/or malignant strictures, CBD), which would be a significant advancement in the field as this could reduce the frequency of invasive procedures undergone by the patient. This would curtail the occurrence rate of potential complications associated with ERCP.
